# An artificial intelligence-integrated analysis of the effect of drought stress on root traits of “modern” and “ancient” wheat varieties

**DOI:** 10.3389/fpls.2023.1241281

**Published:** 2023-10-13

**Authors:** Ilva Licaj, Domenico Felice, Chiara Germinario, Clarissa Zanotti, Anna Fiorillo, Mauro Marra, Mariapina Rocco

**Affiliations:** ^1^ Department of Science and Technology, University of Sannio, Benevento, Italy; ^2^ Department of Management Engineering, Polytechnic of Milan, Milan, Italy; ^3^ Department of Biology, University of Tor Vergata, Rome, Italy

**Keywords:** wheat, osmotic stress, root, artificial intelligence, phytohormones

## Abstract

Due to drought stress, durum wheat production in the Mediterranean basin will be severely affected in the coming years. Durum wheat cultivation relies on a few genetically uniform "modern" varieties, more productive but less tolerant to stresses, and "traditional" varieties, still representing a source of genetic biodiversity for drought tolerance. Root architecture plasticity is crucial for plant adaptation to drought stress and the relationship linking root structures to drought is complex and still largely under-explored. In this study, we examined the effect of drought stress on the roots’ characteristics of the “traditional” Saragolla cultivar and the “modern” Svevo. By means of “SmartRoot” software, we demonstrated that drought stress affected primary and lateral roots as well as root hair at different extents in Saragolla and Svevo cultivars. Indeed, we observed that under drought stress Saragolla possibly revamped its root architecture, by significantly increasing the length of lateral roots, and the length/density of root hairs compared to the Svevo cultivar. Scanning Electron Microscopy analysis of root anatomical traits demonstrated that under drought stress a greater stele area and an increase of the xylem lumen size vessel occurred in Saragolla, indicating that the Saragolla variety had a more efficient adaptive response to osmotic stress than the Svevo. Furthermore, for the analysis of root structural data, Artificial Intelligence (AI) algorithms have been used: Their application allowed to predict from root structural traits modified by the osmotic stress the type of cultivar observed and to infer the relationship stress-cultivar type, thus demonstrating that root structural traits are clear and incontrovertible indicators of the higher tolerance to osmotic stress of the Saragolla cultivar. Finally, to obtain an integrated view of root morphogenesis, phytohormone levels were investigated. According to the phenotypic effects, under drought stress,a larger increase in IAA and ABA levels, as well as a more pronounced reduction in GA levels occurred in Saragolla as compared to Svevo. In conclusion, these results show that the root growth and hormonal profile of Saragolla are less affected by osmotic stress than those of Svevo, demonstrating the great potential of ancient varieties as reservoirs of genetic variability for improving crop responses to environmental stresses.

## 1 Introduction

The projected increase in greenhouse gas emissions is now incrementing the temperature of the Earth. The accelerating pace of climate change, associated with rising global populations and incomes, threatens food security everywhere (Nelson et al., 2009). Among the environmental factors that negatively affect plant growth and development in the global warming scenario, drought is the most critical worldwide (Boyer, 1982; Mittler, 2006; Lipiec et al., 2013; Awasthi et al., 2014). In fact, it can profoundly affect the chemical composition, morphology, and physiological functioning of the plant, ultimately resulting in a severe restriction of crop yield (Seki et al., 2007; Brenchley et al., 2012; Vadez et al., 2012; Verma et al., 2021). Therefore, the study of plant adaptation to drought stress is a crucial research issue for crop production. Wheat (*Triticum aestivum L*.), as the second of the top three primary cereal types (Tilman et al., 2011), is one of the most widely adapted in different growing environments around the world. The situation of wheat production may become more problematic since global warming has significantly affected wheat yields, with losses reaching up to 6.4% for every 1°C of temperature increase (Liu et al., 2016; Miller et al., 2019). The risk of loss of wheat production due to drought stress is expected to increase by almost 12% by the end of the 21^st^ century (Leng and Hall, 2019).

Roots are the essential plant organ for the absorption of water as well as several macro and micronutrients, including nitrogen, silicon, magnesium, and calcium. Osmotic absorption of water and nutrients is drastically limited under drought conditions, a fact that leads to a severe reduction in plant growth and crop yield (Barber, 1995). However, roots are quite plastic organs that can adapt to cope with lack of water in the soil, so that water absorption under drought conditions is limited not only by the physical and mechanical properties of the soil, but is also deeply influenced by changes in the morphological and anatomical traits of roots (Comas et al., 2013). The relationship linking root anatomy and morphology to drought is complex and still largely underexplored. To open up to the possibility of exploiting the anatomical plasticity of the roots to improve tolerance to drought, more focused investigation is needed. Actually, although it is generally true that a deep, widespread, and branched root system is essential for developing drought-tolerant crops (Fukai and Cooper, 1995; Gowda et al., 2011), the key question of which root traits help the most under drought conditions is still open.

Artificial intelligence (AI) is an emerging branch of computer science with great potential to address a variety of complex problems in the modern world. High-throughput analysis methods as well as “omic” approaches applied to biological systems today provide a wealth of complex data that, without proper processing, can lead to misleading conclusions. In plant biology, AI tools have been successfully applied to modulate plant distribution, identify species, to determine diseases and stress status, diagnose nutrient deficiencies, and in agriculture to administer agrochemicals (Soltis et al., 2020). Since AI algorithms are useful in identifying and classifying individual characteristics within a large set of experimental data, they are a promising tool for analyzing mechanisms of plant stress tolerance expression (Fenu and Malloci, 2021). Furthermore, according to a growing body of evidence, AI has also been shown to be very useful for predicting plant responses to stress (Rico-Chávez et al., 2022).

In our study, two AI algorithms have been used to analyze SEM data regarding changes of anatomical traits of roots from two diversely drought-responsive durum wheat cultivars, i.e. the “modern” Svevo variety (less drought tolerant) and the “traditional” Saragolla variety (more drought tolerant) in response to PEG-6000-simulated drought stress. These data have been integrated with root morphological analyses using “SmartRoot” software and related to HPLC analyses of hormonal changes induced by drought stress in roots of the two varieties. The results demonstrated that root architecture and structural traits are accurate indicators of the higher tolerance to drought of the Saragolla cultivar compared to Svevo, and that AI analysis can be effectively applied for predicting the plant response to environmental stress and for inferring the cultivar-stress relationship.

## 2 Materials and methods

### 2.1 Plant growth and treatments

Seeds of the tetraploid *Triticum turgidum ssp durum* cultivars Svevo (Agrisemi Minicozzi, Benevento, Italy), and Saragolla (Mirra Farm, Benevento, Italy) were surface-sterilized in 20% sodium hypochlorite for 20 min, bathed in distilled water six times, and soaked in the dark at room temperature overnight. Seedlings were grown hydroponically in a half-strength Hoagland’s culture solution at 24°C, in a 14h/10/h light/dark cycle. One week later, half of the plants were shifted to the drought-stress conditions, by adding 18% (w/v) polyethylene glycol 6000 (corresponding to estimated-1.0 Mpa osmotic potential), for 5 days, while the other half was used as a control and remained in culture solution without inducing any stress (Peng et al., 2009; Ye et al., 2013). After treatment, the roots were used to perform physiological, anatomical, and morphological investigations and for hormonal content analysis. Five biological replicates of 20 seedlings per cultivar in each condition were used for analysis by the “SmartRoot” software (Regent Instruments Inc., Quebec, Canada) and Scanning Electron Microscopy (SEM).

### 2.2 Sample preparation for scanning electron microscopy

The root samples of both control and treated samples were first rinsed with deionized water under ambient conditions to remove any impurities on the surfaces of the seedlings. Root samples were prepared by a standard method for the preparation of biological specimens following Ensikat et al. (2010). For cross section analysis, twelve-day-old primary roots were cut with a razor in a 0.2 mm long segment from the apical region, and 1 cm long segment from the differentiation root zone (region of maturation). Three to five healthy mature roots per seedling were selected for the cross section analysis. After that, the samples were immediately fixed in formaldehyde solution (2% formaldehyde, 70% ethanol, 5% acetic acid), then dehydrated with ethanol (80%, 90%, 99%). The fixed roots were subsequently dried with liquid CO_2_ in a K-850 Critical Point Dryer (Quorum Technologies, Laughton, UK). Then, double-sided tape was used to mount the samples on aluminum specimen stubs for the characterization via SEM. Before the observations, a layer of gold was sputtered on samples by using a Q150R ES Sputter Coater (Quorum Technologies, UK). The Scanning Electron Microscope consists in a Zeiss EVO 15 HD VPSEM operating at 15 or 20 kV accelerating voltage to record images. Five independent experiments were conducted, analyzing for each experiment about 20 seedlings for cultivar, in each condition. The root images were then analyzed by the Image J software (version 1.52a, NIH, USA). On the basis of the results from the time course experiment, root hair density was subsequently evaluated on the 12^th^ day after germination. Root hair density was determined as the number of hairs in a representative area of the root elongation zone. The results, in this case, were expressed as number per µm^2^ (Maqbool et al., 2022). The length of ten root hairs randomly selected but evenly distributed across the image, from 20 seedlings, was quantified for each cultivar with ImageJ software (Vieira et al., 2007).

### 2.3 Extraction of phytohormones and HPLC analysis

Extraction of abscisic acid (ABA), indol-3-acetic acid (IAA) and gibberellins (GA_3_ + GA_4_) HPLC analyses were carried out essentially as described in Manzi et al. (2015), with slight modifications. Briefly, 1.0 gr of roots were frozen in liquid nitrogen, homogenized with mortar and pestle and extracted in 2.5 mL of methanol. Each extract was cleared by centrifugation at 16 000 g, for 10 min, at 4°C. The supernatant was then concentrated under vacuum to reach a one-tenth of the initial volume. A volume of pure water adjusted to pH 9 was then added to each sample, which was then extracted with an equal volume of ethyl acetate. Aqueous and organic phases were separated by centrifugation at 16 000 g, for 2 min. The lower aqueous phase was adjusted to pH 3, transferred into a new tube and partitioned against an equal volume of ethyl acetate. The upper organic phase was then recovered, completely dried under vacuum and then dissolved in 30 μl of methanol for HPLC analysis.

HPLC analysis was performed on an LC-20 Prominence HPLC system (Shimadzu, Japan) equipped with an LC-20AT quaternary gradient pump, a SPD-M20A photodiode array detector (PDAD), and a SIL-20 AH autosampler (20 μl injection volume). Plant hormones were separated on a Gemini-NX C18 column (250 × 4.5 mm, 5 μm particle size) (Phenomenex, Torrance, CA), assembled with a Security Guard® pre-column (Phenomenex) by using a gradient of acetonitrile containing 0.1% (v/v) trifluoroacetic acid in aqueous 0.1% (v/v) trifluoroacetic acid, at 45°C; acetonitrile ramped from 15 to 30% over 5 min, from 30 to 50% over 5 min, from 50 to 80% over 2 min, and then restoring the starting elution conditions, at a flow rate of 1.5 mL min^−1^. Separated compounds were identified through their retention times, UV spectra and relative literature data by comparison with IAA (12886, Sigma, St Louis, MO), GA_3_ (G7645, Sigma), GA_4_ (G7276, Sigma) and ABA (A1049, Sigma) standards. These standard compounds were also used to build up calibration curves (in the range of 1–100 μg mL^−1^) at specific wavelengths (λIAA = 254 nm; λABA = 254 nm; λGAs = 205 nm). For quantitative analysis, two different amounts of extract from unknown samples were injected in triplicate. Reported values represent the concentration (expressed as μg of hormone per gram of fresh tissues). GA concentration was reported as the sum of GA_3_ and GA_4_ content. The results of independent assays were used for statistical analysis; the mean value ± SD of three independent extractions is provided (Trupiano et al., 2012).

### 2.4 Artificial intelligence methodologies

This study wants to carry out one of the first tests on the use of AI in the field of plant stress, in order to explore the potential of these techniques applied to the world of plant physiology. Using AI tools in this study has a dual purpose: 1. predicting from a given set of stress-weighted features relative to a single observation, the wheat variety being observed, and 2. inferring the type of relationship between the wheat type and the stress occurred (of course, this relationship varies with different types of wheat). Overall, the research goal is to obtain a program capable of recognizing feature patterns with isolated stressed values and weighted on unstressed values (on this, more explanations in the section 3.3). AI tools were preferred, instead of a simple conditional computer program based on statistical analysis, for a purely technical reason. Unlike classical statistical models, the AI models used are non-parametric models: This means that they are structurally independent from the statistical parameters, inevitably conditioned by empirical observations. It is therefore not necessary to verify any parametric assumption which, by definition, could also be statistically non-correct. The use of AI models brings two great advantages: 1. AI models are much more robust than classical statistical models for what has just been said; 2. AI models are well-generalizable tools that cannot be used only in the Saragolla-Svevo case, as they are valid and robust for classifying different, more complex and difficult to distinguish varieties.

As for the technical methodology used to develop the AI tools, an “online” approach was chosen: This means that the choice of AI models did not take place a-priori. Conversely, it was first decided to carry out a preliminary statistical analysis. This solution has allowed to: 1, choose, on the basis of data, the most suitable algorithms for the problem to be solved; 2, understand in clear manner if the final tests on the algorithms chosen are consistent with the processed data.

### 2.5 Data processing

8-bit grayscale images acquired from SmartRoot Analyzer System (Regent Instruments Inc., Quebec, Canada), were analyzed for primary and lateral root length, and lateral root number. Cross sectional and hair root images received from SEM, were analyzed by using ImageJ software (version 1.52a, NIH, USA). The analysis of variance was carried out to determine differences among the treatments and varieties. For comparisons between two groups, statistical significance was calculated using non-paired two-tailed student`s t-test (Graph Pad Prism 5 software). Mean comparisons between more than 2 groups were done using the least significant difference (LSD). Bars are represented as the standard deviation of the mean (SD). p <0.05 was considered the minimum statistically significant. 198 experimental observations spread among five features, respectively: Cortex Cell Area, Stele Cell Area, Late Metaxylem Area, Stele Cross Section Area, Total Cross Section Area. This Dataframe constitutes the starting point for AI development. Specifically, two AI algorithms were used: Logistic Regression and K-Nearest Neighbors classifier (K-NN).

## 3 Results

### 3.1 Effect of osmotic stress on root architectural traits of Svevo and Saragolla cultivars

**Figure 1:**
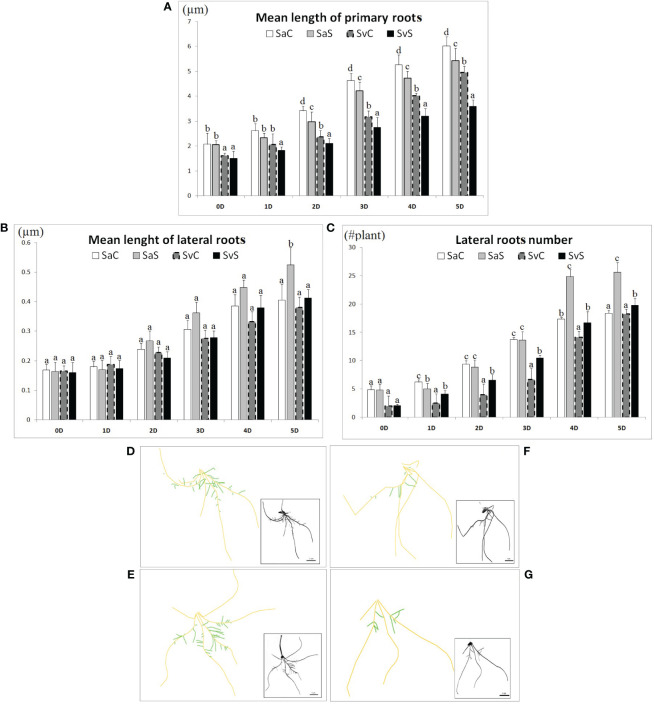
Effect of osmotic stress on morphological root`s traits in the Saragolla and Svevo varieties. Mean length of primary roots (µm) **(A)**; mean length of lateral roots (µm) **(B)**; lateral roots number (#plant) **(C)**. Five-day-old seedlings of both varieties were subjected to osmotic stress treatment with 18% PEG-6000 (w/v) for 5 days and the indicated parameters measured at the reported time points for control and stressed samples. SaC = Saragolla Control, SaS = Saragolla Stressed, SvC = Svevo Control, SvS = Svevo Stressed. Data are presented as mean ± SD. Letters indicate significant differences (p < 0.05) according to LSD test. Panels from **(D–G)**: Tracking of the roots by means of SmartRoot software analyzing 20 seedlings per each condition, number of replicates = 5: **(D)** Saragolla control, **(E)** Saragolla stressed, **(F)** Svevo control and **(G)** Svevo stressed; orange: primary roots; green: lateral roots.

**Figure 2:**
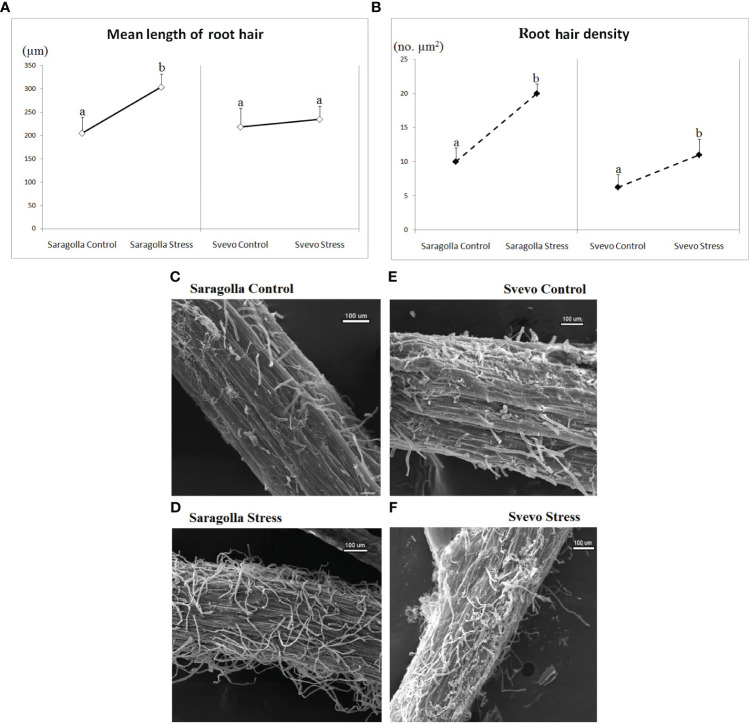
Effect of osmotic stress on root hair length (µm) **(A)**, root hair density (no. µm^2^) **(B)** and root hair images **(C–F)** acquired via Scanning Electron Microscopy. Root hair morphology status was monitored and calculated after 5 days of osmotic stress treatment with 18% PEG-6000 (w/v). Data are presented as mean ± S.D., analyzing 20 seedlings per each condition, number of replicates = 5. Letters indicate significant differences (p< 0.05) according to student`s t-test.

The Svevo and Saragolla seedlings subjected to 18% (w/v) PEG-6000-induced osmotic stress for 5 days showed adaptive alterations in root characteristics, in order to face adverse environmental conditions. Our results revealed significant variations in primary and lateral roots (**Figure 1**), as well as in root hair density (**Figure 2**). A representative example of root images from both cultivars, in control and osmotic stress conditions, acquired with “SmartRoot” software, is shown in **Figures 1D–G**. Treatment with osmotic stress caused a reduction in primary root elongation compared to unstressed samples in both varieties but to a different extent. As shown in **Figure 1A**, at each time point tested, control primary roots grew longer than stressed ones in both cultivars. However, this difference remained practically constant in Saragolla, from 1 to 5 days (~ + 10%, control vs. stressed), while it progressively increased in Svevo (from + 11.6% at 1 day to 27.3% at 5 days, control vs. stressed). The opposite trend was observed in both varieties for the lateral roots, but also in this case it was quantitatively different. In fact, as shown in **Figure 1B**, after an initial reduction (1 day), stressed lateral roots were able to recover their rate of elongation and grew longer than unstressed controls at the end of treatment (5 days). This effect was much more pronounced for the Saragolla cultivar, whose mean lateral root length after 5 days of treatment exceeded that of the control by 29.3%, compared to 8.6% of the Svevo cultivar. A fairly similar trend was observed for the number of lateral roots. As shown in **Figure 1C**, stressed samples of both varieties increased the number of lateral roots, but this effect was much more pronounced for the Saragolla (+ 39.1%, stressed vs. control, 5 days) compared to the Svevo cultivar (+ 8.5% stressed vs. control, 5 days).

Considering that root hairs play an important role in water absorption, we also investigated whether PEG-6000 treatment had any effect on root hair number and length, measuring the root hair density (RHD) and the root hair mean length (RHL), in the root differentiation zone of the two cultivars. As shown in **Figures 2A, B**, a clear difference in the RHL and RHD of root hairs was observed between PEG-6000 exposed and control samples for both varieties, but to a different extent. In the Svevo cultivar, RHD increased by 76% and RHL by 16.3%, after 5 days, respectively. In the Saragolla cultivar these parameters showed a significantly higher increase (+ 100% RHD and +48% RHL after 5 days). In **Figures 2C–F**, a representative example of SEM images of the elongation zones of control and stressed roots of the two cultivars, from which RHD and RHL have been calculated is shown. On the overall, these data demonstrate that the osmotic stress treatment profoundly affected root morphology and that the Saragolla variety had a more efficient adaptive response.

### 3.2 Effect of osmotic stress on root anatomical traits of Svevo and Saragolla cultivars

**Figure 3:**
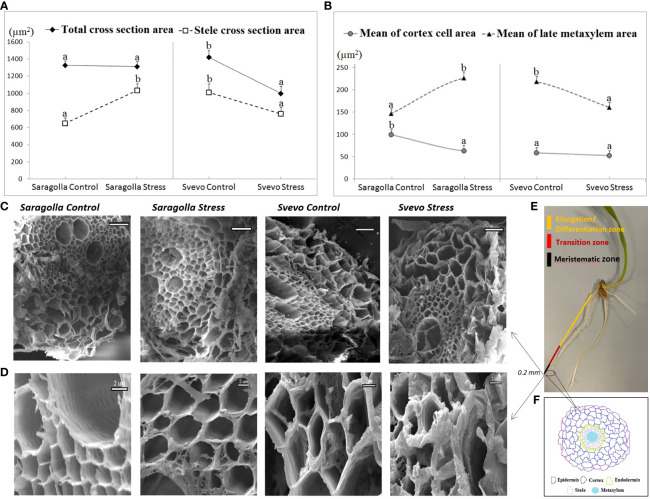
Root total cross sectional area and stele cross section area **(A)**, cortex cell area and late metaxylem area **(B)** (µm^2^) of Saragolla and Svevo wheat cultivars. Analyses were performed after 5 days of osmotic stress treatment with 18% PEG-6000 (w/v), as reported in 2.2 section. Data are represented as mean ± S.D., analyzing 20 seedlings per each condition, number of replicates = 5. Letters indicate significant differences (p < 0.05) according to student`s t-test. SEM images showing root cell layers **(C)** and root cells **(D)** of Saragolla and Svevo cultivars under control and osmotic stress conditions. Illustrations of sectioning positions along the root **(E)**. 0.2 mm segment of root samples from the apical region and 1 cm segment from the differentiation root zone were taken. Schematic sketch of root cell layers **(F)**.

Root anatomical traits profoundly influence water transport, thereby affecting the efficiency of the uptake and distribution of the water to the whole plant (Hazman and Brown, 2018; Mangena, 2018). Xylem vessel features such as number, diameter and area influence axial water conductance while cortical traits affect radial conductance. Larger xylem vessels and thicker roots are usually associated with improved tolerance to drought (Gowda et al., 2011). In this study, sections of control and osmotically-stressed primary roots of the two cultivars (0.2 mm long segment from the apical region and 1 cm long segment from the differentiation root zone (**Figure 3E**) were subjected to SEM observations in order to estimate the mean total cross sectional area, stele cross section area, cortex cell area, and late metaxylem area (Zulfiqar et al., 2020) (**Figure 3F**). We found that Saragolla and Svevo roots had a comparable total cross sectional area under well-watering conditions. However, under drought stress, this feature remained practically unchanged (- 1.6%) in the Saragolla cultivar, whereas it was significantly reduced in the Svevo cultivar (- 29.6%) (**Figure 3A**). The cortex cell area was reduced in both cultivars by drought stress, but to a larger extent in the Saragolla cultivar (37% compared to 10%) (**Figure 3B**). In contrast, the stele cross section area resulted strongly increased (59%) due to osmotic stress in the Saragolla variety, while it was reduced in the Svevo one (- 25%) (**Figure 3A**). Interestingly, in the Saragolla cultivar to the increase of the stele cross section area, a similar increase of the late metaxylem area (55%) corresponded, while in the Svevo variety a decrease was observed (26%) (**Figure 3B**). It should be noted that the late metaxylem tissue is crucial to ensure proper water availability to plants, since water and minerals are absorbed into protoxylem vessels and then transported upwards through early and late metaxylem (Kim et al., 2014). In **Figures 3C, D** representative images of SEM observations of root tissues and cells of the two cultivars under control and drought stress conditions, respectively, are reported.We can even rely and amplify our previous results based on the data analysis using ImageJ, documenting that treated roots showed breakdown or desiccated cells compared to the control group. Furthermore, it is evident from the SEM images that Saragolla cells under osmotic stress were more swollen than Svevo cells (**Figures 3C, D**), confirming that Svevo wheat roots had a more negative prominent response to water stress than Saragolla. It is qualitatively noticeable that water stress produced a more severe damage to the root structure in the Svevo cultivar compared to the Saragolla cultivar. In general, from SEM data it is possible to infer that drought stress had a stronger impact on the integrity of the Svevo root tissues, while in the Saragolla cultivar, a stronger adaptive response occurred, which involved particularly the increase in the late metaxylem area.

### 3.3 Artificial intelligence application

The starting point for the development and application of any algorithm related to the AI area is the Dataframe. In this case, the Dataframe is the set of single experiments conducted in the laboratory, hereafter referred as “observations” in statistic jargon. The Dataframe consists of 198 experimental observations, which are spread among five features, respectively: Cortex Cell Area, Stele Cell Area, Late Metaxylem Area, Stele Cross Section Area, Total Cross Section Area. To these features, a new discrimination feature, called “wheat”, is added with the fundamental task of distinguishing and keeping track of the observed wheat cultivars, Svevo wheat or Saragolla wheat. To achieve this, a binary variable is adopted, which is equal to 0 if the single observation has as object the Svevo wheat, or is equal to 1 if the single observation has as object the Saragolla wheat. The experiment formulation is set on two groups: the control group and the experimental (stressed) group, each of which is for the two types of wheat. To effectively capture all the characteristics of the stress condition, it was decided to calculate the means of the values for each feature of the two control groups, relating respectively to Svevo wheat and Saragolla wheat; then, the means for each feature are hence punctually subtracted from the individual observation values of the experimental groups, relating to both cultivars. In this way, only the components attributable and caused by the stress condition, imposed by the researchers, are isolated. On this Dataframe thus obtained, all the subsequent AI-based techniques” building processes are hinged on. As a first step, preparatory to the proper selection of AI algorithms, a preliminary statistical analysis was performed, defined in the jargon “Descriptive Statistics”. The goal of this analysis is to assess the degree of data overlap and the quality of each feature in order to convey and direct the subsequent choice of the AI algorithms best suited to the specific data being processed.

An initial synoptic description of the dataframe is shown in **Tables 1**, **2**, where the general descriptive statistics are represented relative to Svevo and Saragolla wheatrespectively.

**Table 1:** Dataframe descriptive statistics referred to Svevo wheat.

	Cortex cell area	Stele cell area	Late metaxylem area	Stele cross section area	Total cross section area	Wheat
**count**	99.000000	99.000000	99.000000	99.000000	99.000000	99.0
**mean**	-5.857686	-8.765763	-60.465100	-240.796254	-109.685189	0.0
**std**	5.166052	3.661760	12.662152	71.341766	49.274722	0.0
**min**	-18.550612	-19.548419	-95.639941	-364.890128	-170.831490	0.0
**25%**	-9.075327	-10.321094	-70.159219	-316.967987	-167.328776	0.0
**50%**	-5.854484	-8.662437	-61.238357	-215.309065	-107.670893	0.0
**75%**	-1.570903	-6.743411	-50.020577	-186.447384	-73.437707	0.0
**max**	3.811140	2.923140	-25.250254	-79.907224	1.228477	0.0

Bold values represent the statistical description.

**Table 2:** Dataframe descriptive statistics referred to Saragolla wheat.

	Cortex cell area	Stele cell area	Late metaxylem area	Stele cross section area	Total cross section area	Wheat
**count**	99.000000	99.000000	99.000000	99.000000	99.000000	99.0
**mean**	-36.451594	32.149189	54.654263	368.304304	-14.181400	1.0
**std**	2.834480	3.602009	35.261250	74.715666	24.535743	0.0
**min**	-44.413301	25.624267	-26.650739	233.618025	-92.791615	1.0
**25%**	-38.736706	28.802054	30.258753	305.886967	-24.578456	1.0
**50%**	-35.308962	31.287905	42.267159	354.286742	-14.991323	1.0
**75%**	-34.110505	35.919143	75.988492	442.516280	9.248548	1.0
**max**	-31.982273	39.258693	147.425968	543.810493	19.862024	1.0

Bold values represent the statistical description.

**Figure 4:**
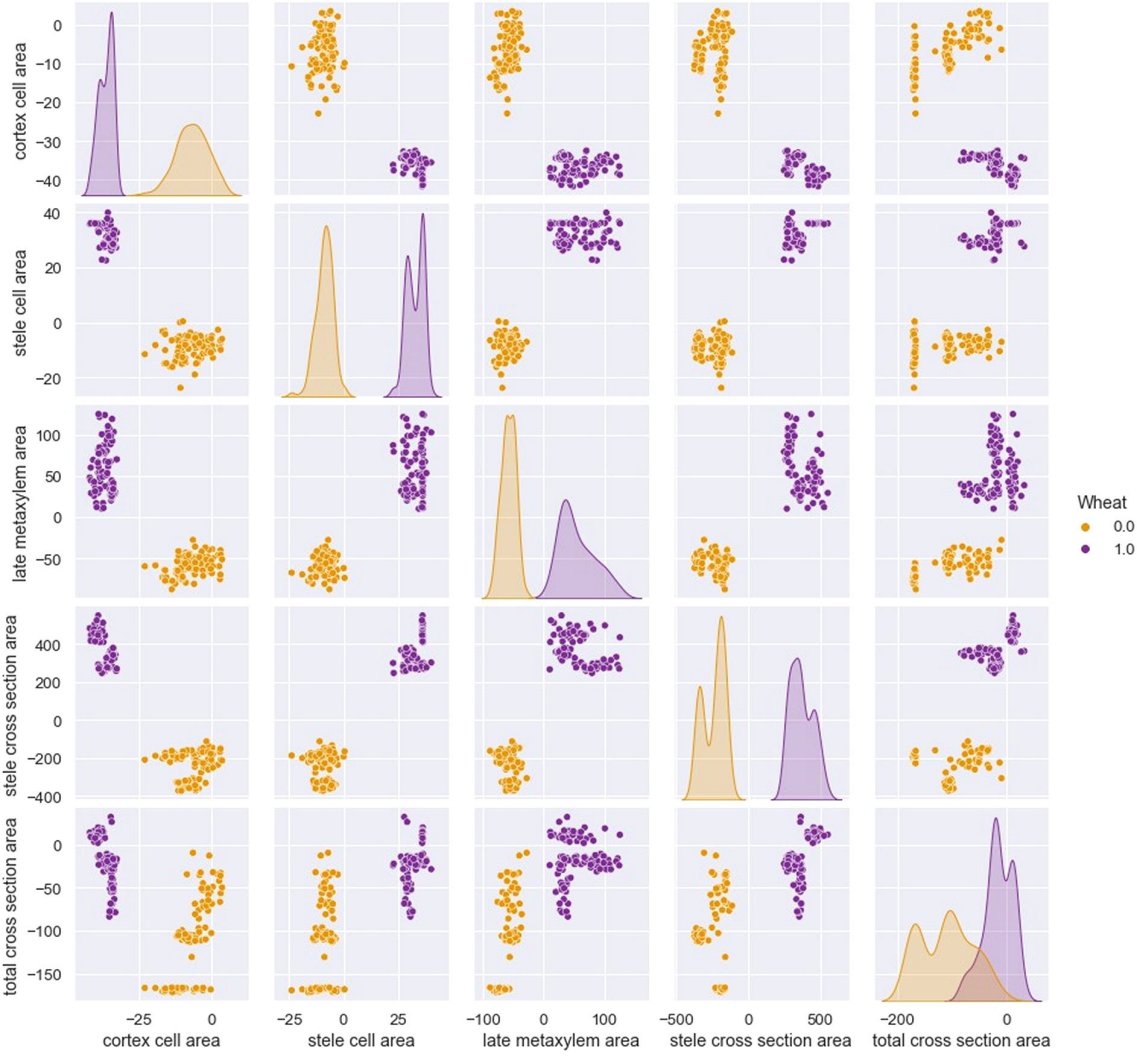
5x5 seized pairplot with “wheat” as discriminating variable:0 for Svevo, 1 for Saragolla (binary variable are used as AI tool does not take as input text variables).

In addition to this, a 5x5-sized pairplot, shown in **Figure 4**, is developed by imposing the type of wheat as the discriminating variable, that is, 0 for Svevo wheat, 1 for Saragolla. On looking at the diagram, it is clear that the differences between the two types of wheat are evident and significantly relevant. Indeed, in each dimension match, the characterization that differentiates the two cultivars is evident. Only in the case of the Total Cross Section Area, there is a slight overlap, which, as it will be figured out hereafter, will not affect the clear data distinction between the two cultivars.

**Figure 5:**
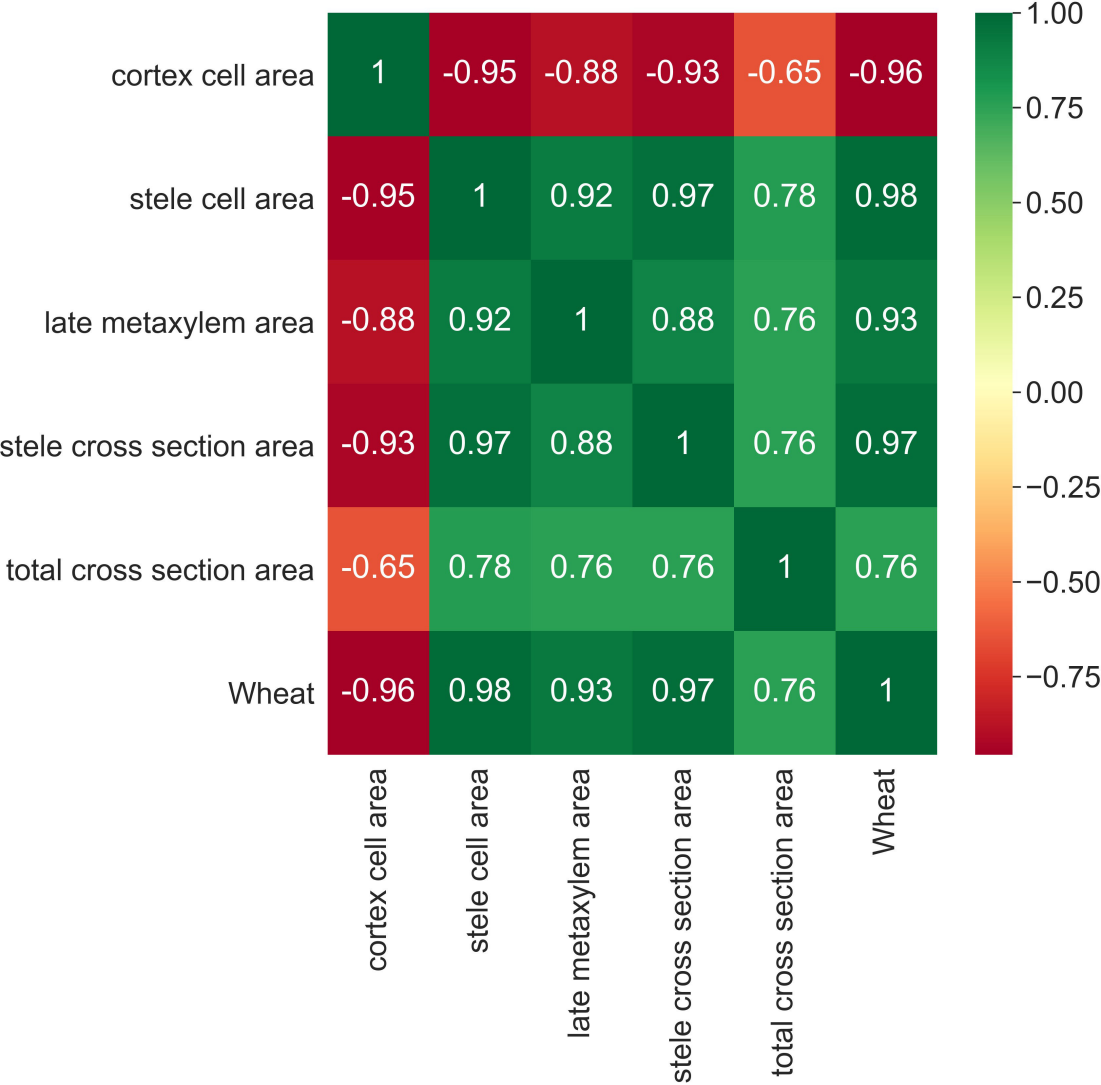
Correlation matrix among variables with color palette ranging from red for negative correlation to green for positive correlation.

Subsequently, a correlation map between all the features is developed, completed also by a heat map, which assumes a gradation of colors ranging from green for positive correlation (+1) to red for negative correlation (-1). The correlation matrix, reported in **Figure 5**, highlights two important aspects of the case under analysis: all variables with the exception of the Cortex Cell Area are positively correlated, with particularly high correlation values; the Cortex Cell Area feature has very high negative correlations with all the other variables.

The results of the Descriptive Statistics convey to proceed with the implementation of specific AI algorithms pertaining to the Machine Learning area, specifically belonging to the Statistical Learning field. Two types of algorithms are chosen: Logistic Regression and K-Nearest Neighbors classifier (K-NN). These two algorithms are relatively simple in formulations: the goal of their choice is to confirm the straightforward and clear-cut distinction in behaviour between the two different wheat types, already traced by the Descriptive Statistics section. For both algorithms, the Dataframe is divided into two parts: 70% of the observations are used for the algorithm training; then, after the training phase, each algorithm is tested on 30% of the remaining observations. The test results will reveal the appropriateness level of the chosen algorithms with respect to the specific data.

As for the Logistic Regression, it is a statistical model used for binomial classification. Logistic Regression is based on a so-called sigmoid function, that is a S-shaped function. This function is used to describe binary probabilities of occurrence as the outcomes are approximated to binary values. Probability modeled through a Logistic Regression is described as follows:


$$p\left(x\right)=\frac{1}{1+e^{-\left({\text{β}}_{0}+{\text{β}}_{1}x\right)}}$$

in which β_0_ is equal to -μ/s, where μ and s are respectively the position and scale parameters, while β_0_ is equal to 1/s.

**Figure 6:**
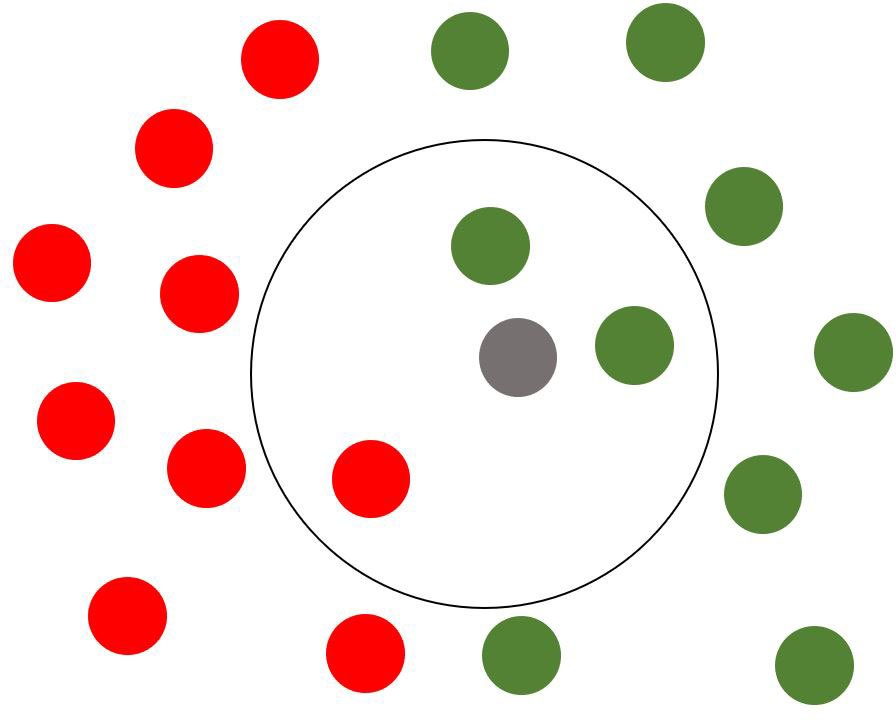
Visual representation of K-NN algorithm’s predicted value assignment process for a new observation.

As for the K-Nearest Neighbors classifier, it is a mathematical model that considers the single observation to be predicted and a number K observation closer to such an observation. The closeness is evaluated through a minimum distance measure: In this case, the Euclidean distance is adopted. Subsequently, the algorithm carries out a weighting of the K distances calculated, rounds the result, and assigns the result found as “predicted” value to the relative observation. A practical representation of the K-NN algorithm is shown in **Figure 6**: The grey point is the observation to be predicted, the red points are observations belonging to the first class, the green ones to the second class. The black circle represents the distance. The grey point will be predicted to belong to the second class (green points) due to the minimum distance rule.

**Figure 7:**
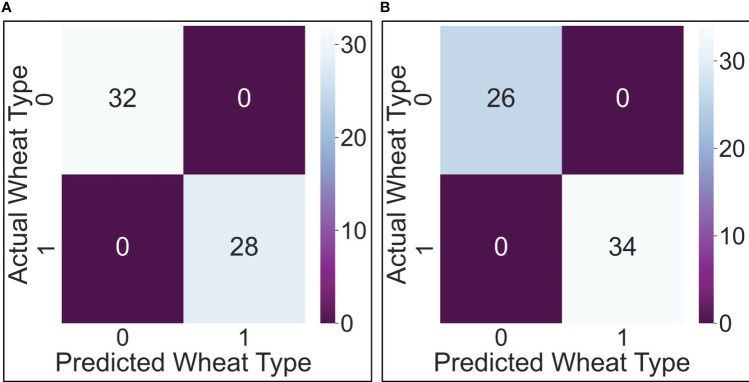
Confusion matrix for the Logistic Regression algorithm **(A)**; Confusion matrix for the K-Nearest Neighbors (K-NN) algorithm **(B)**.

Based on these theoretical formulations, the two algorithms are coded and trained. The development of the AI algorithms takes place in the Jupiter environment, through the Python language. In the testing phase, both algorithms performed very well: 0 false negatives and 0 false positives in both cases. **Figures 7A, B** report the confusion matrixes for the Logistic Regression and the K-Nearest Neighbors, respectively.

Specifically, with reference to the reasons for using AI set out in section 2.4 the algorithms have made possible to pursue both purposes: prediction (i.e., predicting from a given set of stress-weighted features of a single wheat observation, what will be the variety of the cultivar observed), and inference (i.e. inferring the type of relationship between the type of the wheat and the stress occurred).

As for the prediction, both algorithms, despite being relatively simple in their formulation, have perfectly succeeded in predicting the correct type of wheat. This is demonstrated by the confusion matrix shown in **Figure 7**. Such confusion matrix, computed after the test phase of the algorithms, indicates how the algorithms perform: for each algorithm prediction, the software counts the number of correct and incorrect predictions both for positive and negative of algorithms’ outcomes. Overall, the higher are the real positives and negatives with respect to the false ones the better is the algorithms’ performance. In this case, as it emerges from **Figure 7**, both algorithms’ tests show a high number of real positive and real negatives, meaning both algorithms have perfectly succeeded in predicting the correct type of wheat. As for the inference, the types of relationships between the type of wheat and the stress occurred are statistically distinct: this clearly emerges by looking again at **Figure 7** but, this time, at the number of false positives (0) and false negatives (0). This means that the stress response behaviors of Svevo and Saragolla respectively show a clear-cut differentiation between the two types of wheat, confirming that the two relationships “stress-wheat type” are different: this result on false positives and false negatives is not accidental, but it is perfectly explainable from examining **Figure 4**, which shows the distribution of the features. As explained above, the feature values are not absolute values of the stressed group, but are stressed values averaged by the unstressed values, performed for each cultivar. The distribution of values is already distinguishable. The results on the confusion matrix, concerning 0 false negatives and 0 false positives, are perfectly in line with the descriptive statistics.

### 3.4 Effect of osmotic stress on hormone contents in roots of Svevo and Saragolla cultivars

**Figure 8:**
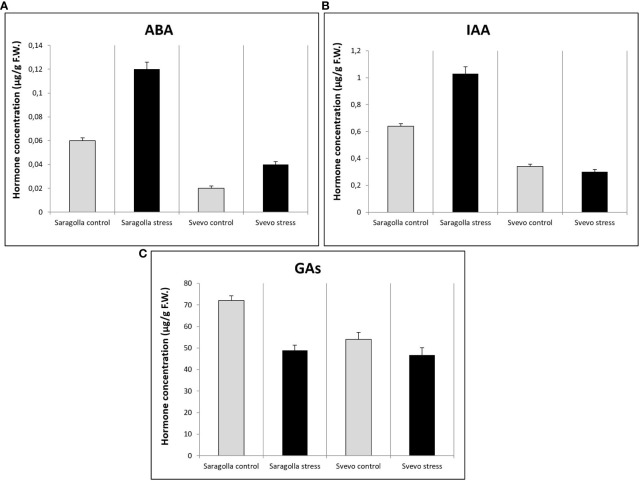
ABA **(A)**, IAA **(B)** and Gas (GA_3_ + GA_4_) **(C)** content (µg/g F.W.) in roots of Saragolla and Svevo seedlings. Analyses were performed after 5 days of osmotic stress treatment with 18% PEG-6000 (w/v). Data are represented as mean ± S.D., number of replicates = 3.

Water stress profoundly influences root morphology and architecture, a fact that underlies extensive transcriptional reprogramming mediated by hormonal signaling and regulation. Therefore, we analyzed the changes in hormone content of Saragolla and Svevo cultivars induced by osmotic stress treatment. The main hormones known to be involved in the response to drought stress, affecting root development were considered, such as primarily, abscisic acid (ABA) and indol-3-acetic acid (IAA), as well as gibberellins (GAs: GA_3_ + GA_4_). The results demonstrated that the roots of the Saragolla cultivar, under well-watering conditions, contained higher amounts of ABA than the roots of the Svevo cultivar (0.06 ± 0.0024 μg/g F.W. vs 0.02 ± 0.002 μg/g F.W.). Treatment with osmotic stress treatment determined an increase in ABA content approximately to the same extent in both varieties (0.12 ± 0.006 μg/g F.W.; +100% Saragolla vs 0.04 ± 0.0024 μg/g F.W.; +100% Svevo) (**Figure 8A**). As far as IAA, the basal levels under control conditions were higher in the Saragolla cultivar (0.64 ± 0.0192 μg/g F.W.) than in the Svevo cultivar (0.34 ± 0.017 μg/g F.W.). The osmotic stress induced a significant increase in IAA amount only in the Saragolla cultivar (1.03 ± 0.051 μg/g F.W.; +60.9%) while in the Svevo cultivar a slight decrease was observed (0.3 ± 0.018 μg/g F.W.; -11.7%) (**Figure 8B**). GAs levels under control conditions were higher in the Saragolla cultivar than in the Svevo cultivar (72.2 ± 2.16 μg/g F.W. vs 54.0 ± 3.24 μg/g F.W.) while the osmotic stress treatment determined a decrease in GA levels in both varieties, although to a higher extent in the Saragolla variety (48.9 ± 2.16 μg/g F.W.; -32% vs 46.8 ± 3.276 μg/g F.W.; -13%) (**Figure 8C**).

## 4 Discussion

Limited water availability strongly affects wheat production in vast areas of the world. A necessary option to mitigate this unfavorable situation is to develop high-productive, drought-tolerant wheat varieties. To this end, “ancient”, that is, local or traditional cultivars, more tolerant to environmental stress than elite varieties used in intensive agriculture, represent a reservoir of genetic diversity that can be exploited to select traits of tolerance.

**Figure 9:**
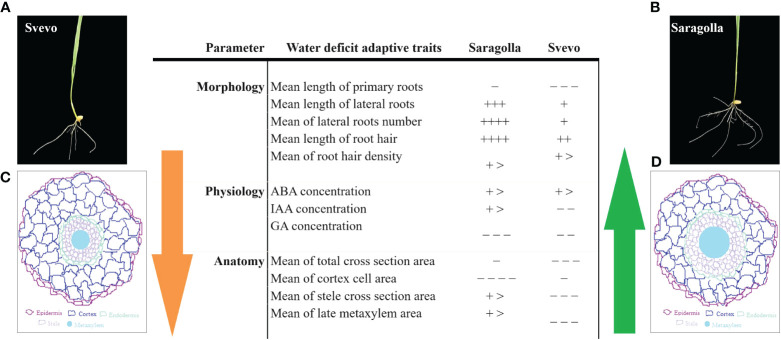
Summary of adaptive changes to PEG-6000 induced osmotic stress occurring in roots of Saragolla and Svevo seedlings. Symbols are as follows: +, increase; −, reduction in water deficit (+/−, less than 10%; ++/− −, more than 10% to less than 20%; +++/− − −, more than 20% to less than 35%; ++++/− − − −, 35%–50%; and more than 50%, + >). ABA, abscisic acid; IAA, indole acetic acid; GAs, gibberellins. (**A, B**)- images of osmotically stressed Svevo and Saragolla cultivars, respectively; (**C, D**)- Schematic picture of root cross section of Svevo and Saragolla cultivars, respectively.

Tolerance to drought is a complex phenomenon, involving genetic, biochemical, physiological, morphological, and structural adaptation. In a previous study (Licaj et al., 2023), we have investigated the effect of PEG-simulated drought stress on the roots of an elite variety of durum wheat, Svevo and a traditional one from South Italy, Saragolla. The effect of PEG-induced osmotic stress on the growth of roots of the two cultivars was analyzed at the biochemical, molecular biology, and proteomic level and allowed the highlighting of molecular determinants of the higher tolerance to osmotic stress of the Saragolla cultivar. In this study, the investigation focused on the effect of PEG-induced stress on the morphological and anatomical characteristics of the roots of the two cultivars. In **Figure 9**, a summary of adaptive changes of key morphological and anatomical traits and of hormone content in response to osmotic stress ascertained by our analysis in Saragolla and Svevo root seedlings is shown.

The remodeling of the root morphology and anatomy following water stress represents the highest level of complexity of the tolerance response and underlies genotype-specific biochemical and physiological mechanisms (Dhanda et al., 2004; Rauf et al., 2007). It is generally accepted that a deep and branched root system is a fundamental component of tolerance to drought. In wheat, root traits are quite sensitive to drought stress (Kumar et al., 2010; Ji et al., 2014) and it has been shown that the plasticity of root morphology influences the growth of the whole plant (MansChadi et al., 2006; Robin et al., 2021). Therefore, a better understanding of the changes in the morphological and structural traits of the root system produced by the water deficit is crucial for developing drought-tolerant crops. In our study, the treatment with PEG caused a reduction in the elongation of primary roots, which was higher in the Svevo variety compared to Saragolla, whereas the osmotic stress increased the length of lateral roots, also in this case to a greater extent in the Saragolla variety. The length and density of root hairs followed the same trend, with an increase under PEG treatment, higher in the Saragolla cultivar. Studies in different species provide evidence that water stress reduces root growth (Westgate and Boyer, 1985; Sharp and Davies, 1989; Spollen et al., 1993), so that the ability to maintain root growth under drought stress is considered an important adaptive trait of plants to increase water uptake (Rodrigues et al., 1995). A study on twenty-two wheat genotypes (Robin et al., 2021) showed that PEG osmotic stress altered root morphology and hair traits of wheat seedlings. The maximum length of main roots generally increased, but to a very variable extent among the genotypes, and two of them showed the opposite trend. The length and density of the first and second order lateral roots also increased, as well as the root hair density of the main and lateral roots. Principal component analysis indicated that the density of lateral roots and root hairs is closely associated with tolerance to osmotic stress. This and other studies (Terletskaya et al., 2020) demonstrate that osmotic stress profoundly influences wheat root morphology in a genotype-dependent way and confirm that the increase in the surface area of young roots is an adaptive strategy to increase water absorption under osmotic stress. Consequently, our results demonstrate that osmotic stress caused a large remodeling of root morphology in the two varieties and that this effect was dependent on the wheat genotype, with the Saragolla variety able to express a better tolerance response to water stress than the Svevo cultivar. As reported by Ji et al. (2014), the possible mechanism underlying the remodeling of wheat root morphology relies on the premature differentiation of the apical meristem of the main roots induced by osmotic stress, which determines the cessation of the growth of primary roots, thus allowing the outgrowth of lateral roots.

In addition, the AI analysis carried out by means of two algorithms of several SEM observations using different anatomical features (total cross sectional area, cortex cell area, stele cell area, stele cross section area and late metaxylem area) of the primary roots of the two cultivars under control and stress conditions, allowed the establishment of a clear-cut correlation between these characteristics and the different expression of tolerance to osmotic stress of the two varieties. PEG treatment induced: i) a significant reduction in total cross-sectional area only in the Svevo variety and of the cortex area in both cultivars, but to a greater extent in Saragolla. ii) an increase in the stele cross section area in Saragolla and a reduction in Svevo. iii) an increase in the late metaxylem area in Saragolla and a reduction in Svevo. Increase of the root diameter under water stress is functional to a better ability of roots to penetrate the soil and to the development of internal structures for water transport (Wu and Guo, 2014; Terletskaya et al., 2020). Stele diameter enlargement contributes to tolerance to osmotic stress by determining higher axial conductivity and reduced radial conductivity (De Bauw et al., 2019; Ouyang et al., 2020). In a previous study on three different wheat species, it was determined that the modulation of root anatomical traits by osmotic stress was species-specific. The increase in root diameter under osmotic stress, although occurring in all varieties, was due to different components (Terletskaya et al., 2020), and the stele radial section area/root radial section area ratio was increased to a greater extent in the more tolerant varieties. In our conditions, while in the Saragolla cultivar osmotic stress determined an increase in the metaxylem and stele area and a decrease in the cortex area, with a substantial invariance of the root cross sectional area, in the Svevo cultivar both the cortex and metaxylem and stele area were negatively affected by osmotic stress, with a decrease in the root cross sectional area.

These results clearly indicate that traits of the anatomical structure of the primary roots were affected with an opposite trend in the two cultivars, with only the Saragolla cultivar expressing a tolerance response to osmotic stress. In this regard, the use of AI algorithms for the analysis of data constitutes a novel approach, still little exploited for the study of plant tolerance to stress. The AI results demonstrate how the behavioral diversity between Saragolla and Svevo wheat under water stress is substantially different and divergent and, therefore, interesting from the point of view of contribution to the academic research. This outcome is further reinforced by the particular selection of algorithms, relatively simple in their formulation and entry-level in the world of AI. This choice was appropriate in the light of the results: the results confirmed how even relatively simple algorithms succeeded perfectly in clearly distinguishing the two behaviors, confirming the crystal-clear difference of behaviors between wheat cultivars. This study allowed to train the AI algorithms on root structural data from SEM observations, in order to recognize the varieties to which a sample belongs. In fact, by submitting relatively few data of a specific sample, the AI algorithms were perfectly able to identify whether the sample in question belonged to the Saragolla or to the Svevo variety. This opens the path for future, interesting, and more powerful applications in the field of plant biology, where more complex algorithms, relative to the areas of Deep Learning and Reinforcement Learning, could be used.

Phytohormones participate in plant responses to environmental stresses, including drought, inducing adaptive changes in metabolism, physiology and of plant architecture, in order to improve survival (Guo et al., 2020). Although it is now evident that root development under physiological conditions and remodeling under stress are the results of the interplay of the action of different hormones (Nishiyama et al., 2011; Shi et al., 2014; Cui et al., 2015; Kumar & Verslues, 2015; Rowe et al., 2016; Ullah et al., 2018), detailed information is quite limited, especially in cereal crops (Krugman et al., 2011; Ptošková et al., 2022). In wheat, although abscisic acid (ABA), as in most of the studied species, is the main hormone regulating tolerance to drought (Lata and Prasad, 2011), recent evidence points to a role of other hormones, including two general regulators of plant growth, such as auxin (IAA) and gibberellins (GAs) (Tanimoto, 2012; Coelho et al., 2013; Ptošková et al., 2022). ABA is the major stress signal from roots to shoots and ABA signaling in roots, together with modulation of IAA synthesis and transport, remodels root morphology and anatomy, to maximize water uptake under unfavorable conditions (Karlova et al., 2021). Auxin plays a crucial role in different aspects of root development from embryo to mature plant (Overvoorde et al., 2010) and is a key regulator of lateral roots growth, which under water stress are major determinants of water uptake efficiency (Péret et al., 2009). Gibberellins stimulate the growth of most plant organs through cell enlargement and cell division (Colebrook et al., 2012; Nelissen et al., 2012) and it has been reported that reduced GAs levels enhance drought tolerance in different species (Ullah et al., 2018), including wheat (Ptošková et al., 2022). Taking into account the increasing evidence concerning the roles played by these hormones in the remodeling of wheat roots under water stress, we performed the analysis of the changes of ABA, IAA and GAs content caused by PEG treatment in roots of the two cultivars, to highlight a possible differential hormonal regulation. The results showed that the Saragolla roots contained a significantly higher amount of ABA under control conditions than the Svevo cultivar and that osmotic stress determined an increase in ABA concentration in both cultivars. The elongation of primary roots at low water potential is dependent on the accumulation of ABA (Spollen et al., 2000) and it has been shown that ABA modulates IAA transport in the root tip, thereby stimulating root growth under water stress (Xu et al., 2013). Lateral roots development under stress conditions greatly impacts the ability of the plant to maintain water uptake. The results showed that the IAA concentration was higher in Saragolla under control conditions and was increased by osmotic stress only in the Saragolla cultivar. Lateral root formation and development strictly depend on auxin biosynthesis and transport in the root (Vanneste et al., 2005; De Smet et al., 2007; Okushima et al., 2007; Moreno-Risueno et al., 2010) and it has been shown that ABA influences lateral root development by interfering with IAA homeostasis (De Smet et al., 2003; Deak and Malamy, 2005; De Smet et al., 2006; Duan et al., 2013; Osakabe et al., 2013). Recent evidence (Devaiah et al., 2007; Ding et al., 2015) has shown that WRKY factors promote lateral root formation during osmotic stress by regulating the ABA/IAA cross-talk in the root. In a previous study (Licaj et al., 2023) we have shown that different wheat WKRY factors involved in the response to abiotic stress were up-regulated by osmotic stress to a much higher degree in the Saragolla cultivar than in the Svevo cultivar. Xylem formation, development and remodeling by stress are modulated by different hormones, among which IAA plays a pivotal role (reviewed in Motose et al., 2001; Fukuda et al., 2007). This evidence is in good accordance with the increase of the metaxylem area induced by osmotic stress only in the Saragolla variety, where IAA concentration was also increased. GAs concentrations were higher in the Saragolla cultivar and were decreased by osmotic stress in both varieties, but to a higher extent in the Svevo cultivar. Plants with reduced GAs content have been shown to be more resistant to abiotic stress, including drought (Colebrook et al., 2012). Root growth requires lower concentrations of GAs than shoot growth (Tanimoto, 2012) and exceeding GAs concentration may be inhibitory (Inada and Shimmen, 2000; Coelho et al., 2013), therefore lowering GAs content in roots under water stress may be a mechanism to redistribute growth between shoot and root (Ptošková et al., 2022).

These results in general show that ABA, IAA and GAs levels were generally higher in the Saragolla variety, that osmotic stress determined a significant alteration of hormone concentrations in both cultivars, and that the profile of alteration in the Saragolla cultivar was consistent with the expression from a morphological and anatomical perspective of a more robust response to osmotic stress than the Svevo cultivar.

## Data availability statement

The raw data supporting the conclusions of this article will be made available by the authors, without undue reservation.

## Author contributions

Conceptualization, data curation, formal analysis, investigation, validation, visualization, IL and MR; writing—original draft, IL; methodology, IL, DF, CG, CZ and AF; validation, visualization, writing—review and editing, IL, MM and MR; Supervision, MR. All authors contributed to the article and approved the submitted version.
